# Cerebellitis in systemic lupus erythematosus: an elusive face of neurolupus

**DOI:** 10.1055/s-0046-1827161

**Published:** 2026-08-13

**Authors:** Leonardo Furtado Freitas, Jeffrey S. Ritter, Charif Sidani, Igor Mikityansky, Kevin J. Abrams

**Affiliations:** 1 Baptist Health South Florida, Radiology Associates of South Florida, Department of Radiology, Miami FL, United States.; 2 Florida International University, Herbert Wertheim College of Medicine, Miami FL, United States.; 3 The Center for Arthritis & Rheumatic Diseases, Miami FL, United States.

**Figure 1:**
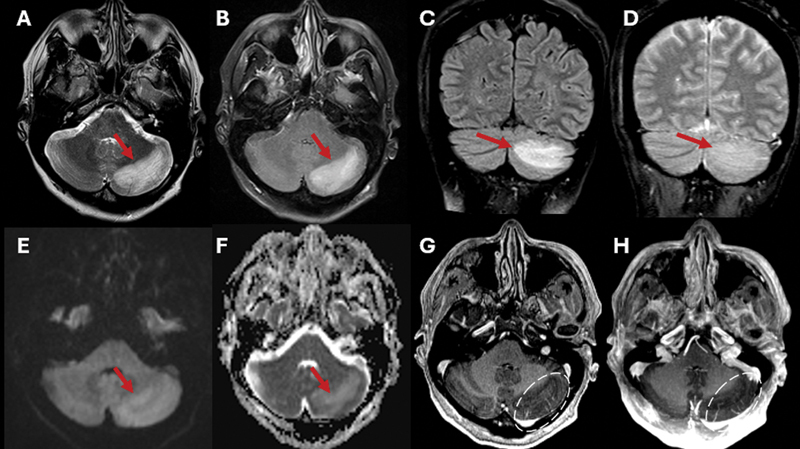
Initial brain magnetic resonance imaging (MRI) scan. ( **A** ) Axial T2-weighted sequence; ( **B** ) axial fluid-attenuated inversion recovery (FLAIR) image with fat suppression; ( **C** ) coronal FLAIR with fat suppression; ( **D** ) coronal gradient-echo imaging; ( **E** ) axial diffusion-weighted imaging (DWI); ( **F** ) axial apparent diffusion coefficient (ADC) map; (G) axial volumetric T1-weighted magnetization-prepared rapid gradient-echo (MPRAGE) postgadolinium imaging; and ( **H** ) maximum-intensity projection (MIP) reformatted image. Extensive corticosubcortical lesion in the left cerebellar hemisphere (red arrows), with facilitated diffusion and no microhemorrhages or calcifications. Prominent local leptomeningeal vascularity noted (dashed white circles).

We herein report the case of a 54-year-old woman with systemic lupus erythematosus (SLE) who developed multifocal, asynchronous cerebellar lesions. An initial brain magnetic resonance imaging scan and a follow-up scan performed 1 week later ( Figures 1 2 ) showed a large left cerebellar lesion with hyperemia and spectroscopic evidence of membrane breakdown. Months later, a new right cerebellar lesion emerged ( Figure 3 ). The cerebrospinal fluid (CSF) analysis revealed neutrophilic pleocytosis, elevated protein, and increased levels of immunoglobulin G (IgG), with negative cultures. Imaging evolution under high-dose corticosteroids and immunosuppression demonstrated complete resolution ( Figure 4 ). Lupus cerebellitis 1 2 3 is rare, and it may mimic infection or a neoplasm. The proposed mechanisms include vasculitis, ischemia, antibody-mediated neuronal dysfunction, and blood–brain barrier disruption. The case herein reported underscores the importance of integrating MRI, CSF analysis, and clinical response for accurate diagnosis.

**Figure 2:**
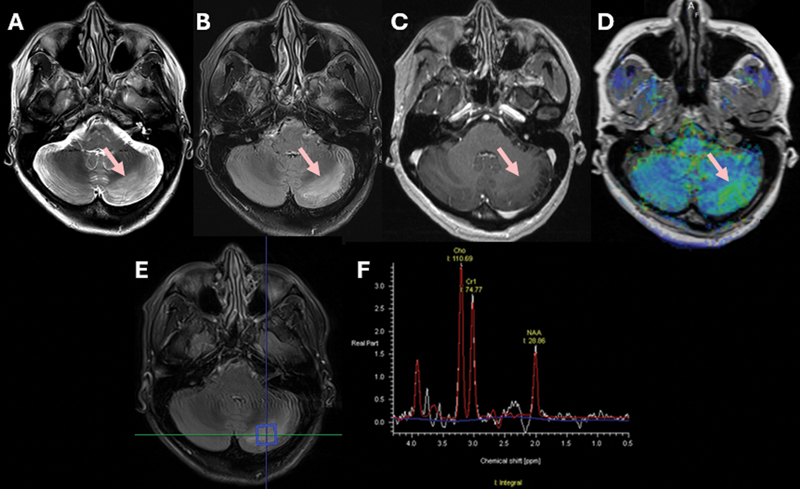
One-week follow-up brain MRI scan. ( **A** ) Axial T2-weighted sequence; ( **B** ) axial FLAIR image with fat suppression; ( **C** ) axial volumetric T1-weighted MPRAGE postgadolinium imaging; ( **D** ) cerebral blood volume (CBV) perfusion map; ( **E,F** ) single-voxel MR spectroscopy with intermediate echo time. Partial involution of the left cerebellar lesion (pink arrows), with decreased enhancement. Increased local blood volume, suggesting hyperemia. Proton spectroscopy with elevated choline (Cho) peak and reduced N-acetylaspartate (NAA) peak, with inversion of the ratios relative to creatine (Cr), consistent with membrane breakdown and loss of neuronal viability/density, respectively.

**Figure 3:**
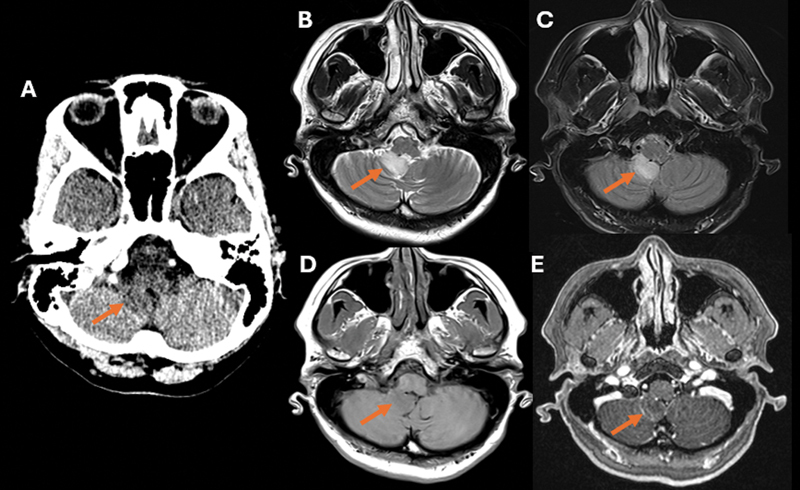
Neuroimaging follow-up after 7 months. ( **A** ) Axial computed tomography (CT) scan, soft tissue window; brain MRI scan showing ( **B** ) axial T2-weighted sequence; ( **C** ) axial FLAIR image with fat suppression; ( **D** ) axial T1-weighted sequence; and ( **E** ) axial volumetric T1-weighted MPRAGE postgadolinium imaging. Interval smaller lesion with similar characteristics in the right cerebellar tonsil (orange arrows).

**Figure 4:**
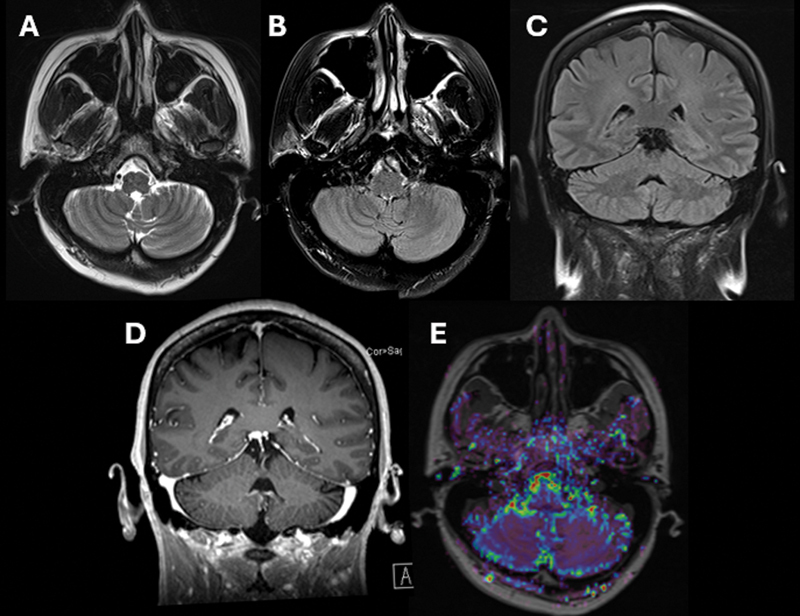
Follow-up brain MRI scan performed a few months later. ( **A** ) Axial T2-weighted sequence; ( **B** ) axial FLAIR image with fat suppression; ( **C** ) coronal FLAIR image with fat suppression; ( **D** ) coronal volumetric T1-weighted MPRAGE postgadolinium imaging; and ( **E** ) CBV perfusion map. Complete resolution of the bilateral cerebellar lesions, without significant sequelae. No evidence of blood–brain barrier dysfunction or perfusion abnormalities.

A chronic autoimmune disease with heterogeneous clinical manifestations and a highly-variable global epidemiology, the incidence of SLE ranges from approximately 1.5 to 11 cases per 100 thousand person-years, and prevalence varies widely, depending on geographic region and ethnicity. The condition disproportionately affects women and is associated with increased morbidity and mortality compared with the general population. 4 Neuropsychiatric involvement represents one of its most complex and heterogeneous manifestations. Therefore, the recognition of lupus-related cerebellitis is essential to ensure an accurate diagnosis and appropriate immunosuppressive treatment.
